# A scoping review of intersectional health research related to the COVID-19 pandemic in North America: key findings

**DOI:** 10.1186/s12889-025-25056-2

**Published:** 2025-10-31

**Authors:** Bukola Salami, Mia Tulli-Shah, Idil Ali, Jonah Zwaigenbaum, Shirley Anne Tate, Hsun-Hui Tseng, Reiko Ogawa, Jaqueline Gahagan, Maud Perrier, Aloysius Nwabugo Maduforo

**Affiliations:** 1 Department of Community Health Sciences, Cumming School of Medicine,, University of Calgary, Calgary, AB Canada; 2 Faculty of Nursing, University of Alberta, Edmonton, Canada; 3 Department of Sociology, University of Alberta, Edmonton, Canada; 4 Department of Taiwanese Literature, National Cheng Kung University, Tainan City, Taiwan; 5 Graduate School of Social Sciences, Chiba University, Chiba, Japan; 6 Department of Gender, Society, and Health, Mount Saint Vincent University, Halifax, NS Canada; 7 School of Sociology, University of Bristol, Bristol, United Kingdom

**Keywords:** Intersectionality, Intersectional research methods, COVID-19, Pandemic, North america, Scoping review

## Abstract

**Background:**

This scoping review maps the key findings of intersectional research related to the COVID-19 pandemic in North America. Intersectional approaches highlight how overlapping systems of oppression shape health and social outcomes.

**Methods:**

A total of 21 studies were included, comprising 10 quantitative, 8 qualitative, and 3 mixed-methods designs. Studies were reviewed to assess the use of intersectional research methods and to identify common findings across the literature.

**Results:**

Intersectional research methods are increasingly utilized in pandemic-related studies in North America. Thematic analysis revealed five key themes: deepening disparities in health care systems, barriers to accessing social services, changes to working conditions across economic sectors, impacts of lockdown restrictions, and impacts on mental health. This review also found that interruptions to community connections influenced access to resources, shaping life chances for some populations. Importantly, intersectional research related to the pandemic has often decentralized race, which contrasts with broader non-intersectional studies.

**Conclusions:**

Findings underscore the need for public health policies informed by intersectional frameworks. Inequities related to class, race, and gender highlight the importance of disaggregated data collection as standard practice. Targeted interventions, such as workplace protections for racialized women in precarious jobs, are critical to addressing compounded vulnerabilities and ensuring equity in pandemic responses.

## Introduction

The COVID-19 pandemic has exposed and exacerbated health and social disparities across the globe. Within North America, the uneven distribution of its impacts has highlighted how intersecting systems of oppression, such as racism, sexism, classism, and ableism—affect individuals and communities differently. Intersectionality, a framework originally conceptualized by Kimberlé Crenshaw [1], provides a critical lens to understand these dynamics by examining how overlapping social identities shape experiences and outcomes within broader structures of power. Building on the work of Crenshaw, scholars like Patricia Hill Collins [2, 3] have further developed the concept, emphasizing the structural, relational, and context-specific nature of intersecting oppressions.

Intersectionality has since evolved as both a theoretical and methodological tool for analyzing disparities. Methodologically, operationalizing intersectionality involves not only using standard methods to explore intersecting social identities but also adapting these methods to critically analyze how structures of power and oppression interact to shape disparities. It moves beyond single-axis analyses, which often isolate variables such as race or gender—to reveal how overlapping social positions collectively influence individuals’ access to resources, opportunities, and health outcomes [2]; Crenshaw [1],. For example, at the micro level, an individual’s experience of healthcare may be shaped by their combined identities as a low-income, immigrant woman of color. This involves analyzing how specific social identities intersect in ways that affect access to care and treatment outcomes. At the meso level, intersectionality reveals how organizational practices, such as workplace policies, create barriers or opportunities that disproportionately impact groups like Black women or gender-diverse individuals [4], providing a lens to critique and refine institutional structures. At the macro level, intersectionality examines how systemic structures like colonialism and patriarchy interact with public health policies to exacerbate disparities during a pandemic [2, 3], emphasizing the need for systemic reforms.

This multi-level understanding of intersectionality guided the scoping review process, informing both the inclusion criteria and the analysis of findings. Researchers aimed to identify and map the key findings of studies that conducted analyses of multiple intersecting social locations within the context of the COVID-19 pandemic in North America. By focusing on results and methods among included studies, this review captures how intersectional analysis has been methodologically operationalized, including adapting standard research techniques to critically examine structural inequities and compounded vulnerabilities.

The COVID-19 pandemic has magnified pre-existing inequities across multiple domains, including healthcare, economic stability, and social services [5, 6]. Marginalized groups have faced higher rates of infection, hospitalization, and mortality, as well as significant economic and psychological burdens [5–7]. These disparities are not random but rooted in systemic inequalities that intersect across social locations such as race, gender, socioeconomic status, and migration status [1, 2].

Despite the urgency of addressing these issues, much of the existing pandemic-related research has adopted non-intersectional approaches, often analyzing single variables in isolation. Such approaches risk oversimplifying complex realities and obscuring the compounded vulnerabilities faced by individuals at the intersections of multiple identities. This gap underscores the importance of intersectional methodologies in uncovering nuanced patterns of inequality and informing equitable policy responses.

This paper presents findings from a scoping review of intersectional research related to the COVID-19 pandemic in North America. For example, included studies explored intersections such as race and gender, class and migration status, or Indigeneity and sexuality to reveal compounded vulnerabilities during the pandemic. It focuses on studies that operationalize intersectional methods to examine at least two intersecting social locations. The review provides critical context for understanding how the pandemic has deepened systemic inequities and highlights the importance of intersectional approaches in shaping more equitable public health and policy responses.

The findings presented here are part of a larger review examining how intersectional research methods were operationalized in pandemic studies. While that analysis will be detailed in a separate paper, this review focuses on the substantive results of the included studies. In doing so, it provides a critical context for understanding the unique challenges faced by marginalized populations during the pandemic and underscores the value of intersectional approaches in shaping more inclusive public health and policy interventions.

## Methods

Researchers drew on Arksey and O’Malley’s (2005) five-step process for conducting scoping reviews [8]. This process enabled us to identify and map key findings, as well as important trends and gaps in knowledge surrounding intersectional research about the COVID-19 pandemic in North America. Through this process, researchers first identify one or more research questions. Next, they identify relevant studies before selecting those which meet inclusion criteria. Third, studies are chosen for inclusion. Fourth, they chart the data. Finally, researchers collate, summarize, and report their findings.

### Stage 1: Identifying the research question

As the first step in this review, researchers identified the following:

1. What are the key findings of research studies that conducted analysis of multiple intersecting social locations in the context of the COVID-19 pandemic in North America?

### Stage 2: Identifying relevant studies

In consultation with the rest of the team, a researcher librarian designed our search strategy. This strategy began with the development of search queries based on subject headings, and key words. Subject headings were then tailored to each specific database searched and included the concepts related to intersectionality that were of most interest to the research team. Subject headings included religion, migration, race, ability, class, sexuality, and ageism in combination with terms related to COVID-19. Researchers used COVID-19 search filters developed by the Canadian Agency for Drugs and Technologies in Health (CADTH).

We searched the following databases: Ovid MEDLINE(R), Ovid Embase, Ovid APA PsycInfo, Ovid Global Health, CINAHL (EBSCOhost), Anthropology Plus (EBSCOhost), EconLit (EBSCOhost), Gender Studies (EBSCOhost), Legal Source (EBSCOhost), LGBTQ + Source (EBSCOhost), Political Science Complete (EBSCOhost), SocINDEX (EBSCOhost), PAIS Index (Proquest), Sociological Abstracts (Proquest), ProQuest Dissertations & Theses Global, Web of Science Core Collection (Science Citation Index, Social Sciences Citation Index, Arts & Humanities Citation Index, Conference Proceedings Citation Index-Science, Conference Proceedings Citation Index-Social Sciences & Humanities, Book Citation Index, Emerging Sources Citation Index), Scopus, HeinOnline, and WHO COVID-19 Global Literature on Coronavirus Disease.

### Stage 3: Selecting studies

**Fig. 1:**
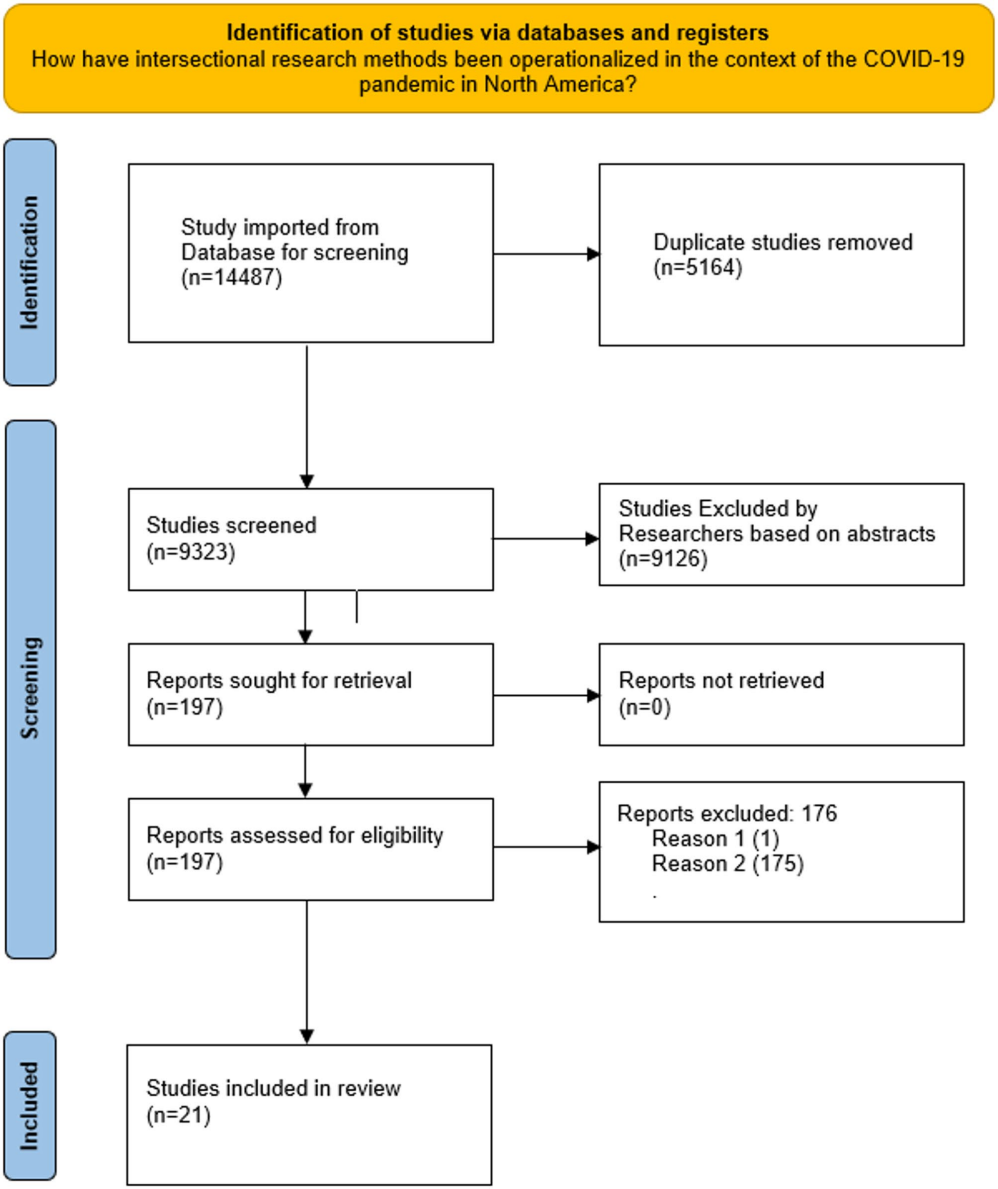
PRISMA Diagram^1^

The database search retrieved a total of 14,487 articles. Of these, 5,164 duplicates were removed during the initial screening process. The abstracts of the remaining 9,323 articles were screened independently by two researchers to determine relevance to the research question. Articles were excluded if they lacked a focus on intersectional methods, did not analyse multiple intersecting social identities (e.g., race, gender, class), or were not related to health-related contexts within the COVID-19 pandemic. Any conflicts during the screening process were resolved by a third team member. Following abstract screening, 197 articles were selected for full-text review. An additional 176 articles were excluded after full-text screening due to irrelevance, lack of intersectional focus, or insufficient methodological detail. This process resulted in a final set of 21 studies for inclusion. For further information, see Fig. 1: Preferred Reporting Items for Systematic Reviews and Meta-Analyses (PRISMA) Diagram.

### Stage 4: Charting the data

There were a total of 21 articles included in this data set. Three researchers extracted data from these articles. Extracting data involved charting author(s) names, year of publication, intersectional social locations under analysis, study purpose, study population, methods, key results, implications, and the ways in which each study engaged with intersectional research methods of oppression, comparison, relationality and reflexivity, complexity, and deconstruction.

### Stage 5: Collating, summarizing, and reporting the results

Included articles were analyzed using thematic analysis, as outlined by Braun and Clarke [9]. This process involved six stages: (1) Data Familiarization, where researchers immersed themselves in the data by reading and re-reading included studies; (2) Generating Initial Codes, identifying patterns and relevant features systematically across the dataset; (3) Searching for Themes, where codes were grouped into broader themes reflecting significant findings; (4) Reviewing Themes, ensuring themes were coherent, distinctive, and relevant to the research questions; (5) Defining and Naming Themes, clearly articulating each theme to capture its essence; and (6) Writing Up, synthesizing the themes and contextualizing them within the study’s intersectional framework.

The coding process was conducted iteratively, with researchers independently coding the data to identify patterns and relevant features. Regular team discussions were held to resolve differences, ensuring consistency and accuracy in the analysis. Reflexivity was embedded throughout the process to critically evaluate researcher biases and their potential impact on theme development [9, 10]. These iterative and collaborative steps ensured greater transparency and methodological rigour, allowing for the identification and synthesis of patterns of compounded vulnerabilities and structural inequities related to the COVID-19 pandemic.

## Findings

A total of 21 studies were included in this scoping review, comprising 10 quantitative, 8 qualitative, and 3 mixed-method studies. Table 1 summarises the study types, methods used, and the associated studies, along with the percentage distribution of each category. Quantitative studies primarily relied on surveys, while qualitative studies frequently utilised interviews. Mixed-method studies combined various approaches such as interviews, surveys, and crowdsourced data. Table 1 provides an overview of the diversity in methodological approaches across the included studies.

**Table 1:** Summary of included studies by study Type, Methods, Count, and percent

Study Type	Count	Percent (%)	Methods Used	Studies
Quantitative	10	47.6%	Survey (7), Secondary Analysis of Survey Data (3)	Allen et al., [11–20]
Qualitative	8	38.1%	Interviews (6), Focus Groups (1), Open-ended Surveys (1)	Bibi [21, 22],; Cardenas-Marcelo et al., 2022; Fortune et al., [23–27]
Mixed Methods	3	14.3%	Interviews, Surveys, Crowdsourced Data	Bettinger-Lopez et al., [28–30]

The most common method used by quantitative studies was survey as seven studies of the 10 used this method [11, 13–15, 18–20]. The other three quantitative studies conducted secondary analysis of survey data collected previously [12, 16, 17]. The majority of qualitative studies used interviews [21, 22]; Cardenas-Marcelo et al., 2022; Fortune et al., [24, 25, 27]. One qualitative study used both focus groups [26], and another open-ended surveys [23]. The three mixed method studies used a mix of interview, survey, and crowdsourced data (Bettinger-Lopenz et al., 2022), interview and survey [29], and both open-ended and closed surveys [30].

Thirteen of the 21 included studies explicitly discussed using an intersectional analysis. Seven of the qualitative studies did so [21, 23]; Cardenas-Macelo et al., 2022; Fortune et al., [24–27]. Additionally, all three mixed method studies [28–30], and three quantitative study also explicitly engaged with intersectionality [16–18]. The other eight studies did not expressly utilize intersectional methods but did analyze the intersections of at least two categories.

All included studies investigated intersections of gender and race, except for Cardenas-Marcelo et al. (2021) who looked to intersections of gender and class. While five of these studies focused analysis only on gender and race [11, 12, 15, 18, 27], others considered gender and race alongside other axes. Five considered intersections of class with gender and race [19–21, 24, 25, 30], and three looked at sexuality, gender, and race [13, 14, 23]. Other intersections under focus included citizenship, class, and race [16], gender, race, class, migration, and sexuality [28], gender, race, class, and migration status [22], class, race, education, and family status [17], gender, race, class, and ability [26], and gender, race, Indigeneity, and sexuality [29].

### Themes

Thematic analysis uncovered five themes across included study findings. This analysis shows that intersectional research on the impacts of the COVID-19 pandemic report reported deepening disparities in health care systems, access barriers to social services, changes to working conditions, lockdown restrictions impacts, and impacts on mental health.

#### Deepening disparities in health care systems

A total of five studies reported findings that suggest pre-pandemic health care system disparities at intersections of gender, race, age, education, and income have deepened since the onset of COVID-19. These studies all note dangers of dis- and mis-information and while calling for large scale communication efforts to better spread accurate information and combat conspiracy theories [11–13, 20, 24]. For example, survey data from two studies revealed that younger women were significantly more likely to search for COVID-19 information when compared to older adults and younger men [12], and white women were more likely than Black women to know where to seek out a COVID-19 test despite experiencing less impacts of COVID-19 [20]. Additionally, Fortune et al. [24] found low-income mothers in rural areas may face inadequate internet access which may negatively impact their capacity to access accurate information and support their families’ well-being during the pandemic. Further, survey data collected in the United States shows women with low socioeconomic status who have less education are more likely to be vaccine hesitant or resistant.

 [11].

Consequently, there is a need for targeted COVID-19 vaccine safety plans to foster trust and address vaccine concerns [11]. The need for targeted communication was also echoed by Card et al., [13] in a quantitative study in Canada showcasing the intersection of sex, gender and income in the greater perceived difficulty of attaining help for drug use among queer men who expressed a desire to seek help during the pandemic. Barriers to accessing information and resources among queer men was associated with lower incomes and having a lack of access to primary healthcare providers to write referrals due to low income [13].

#### Enhanced barriers to social services

A total of five studies concerned shifting social service barriers throughout the pandemic (Bettinger-Lopez & Montes, 2022; Callander et al., 2021; Chaiton et al., [14]; Etowa et al.m 2021; Fortune et al., 2021). Both Bettinger-Lopez & Montes (2022) and Callander et al. (2021) found that barriers to social services changed and intensified during the pandemic. Bettinger-Lopez & Montes (2022) used an intersectional gender lens to highlight the ways that lockdown restrictions exacerbated existing inequities and forced some, especially women without legal status, into cycles of dependence. Many of those who were already excluded from social services were forced to rely on abusive partners for basic human necessities. Focused on sex workers instead of those without status, Callander et al. (2021) found that sex workers were also excluded from government benefits during the pandemic, likely connected to prior marginalization of this labour sector.

Additionally, Fortune et al. [24] considered the intersections of gender, focusing on the impact of class among single mothers and the disadvantages produced as it intersected with race and gender. The complexity of accessing services in rural areas in the context of the pandemic also considered lockdown measures and school closures [24]. Single mothers were found to face financial disadvantages during the pandemic as not having a partner to divide resources forced mothers to prioritize necessities over entertainment and accessing leisure centers for children [24].

In addition to social services supporting basic needs, lockdowns intensified educational barriers for 2SLGBTQ + youth [14]. The researchers used a multinomial logistic regression model to compare demographic correlations [14]. Despite not using an intersectional framework or making note of the dynamic of intersecting identities of sexual and gender minority youth in accessing mental health resources, the researchers used a post hoc analysis in this quantitative study in the US to compare demographic distinctions in access to services. Education was the only association found as significant in barriers to access to social services, with college or university educated 2SLGBTQ + youth less likely to experience barriers in comparison to less educated 2SLGBTQ + youth [14].

#### Changes to working conditions

A total of seven studies found changes to working conditions during the COVID-19 pandemic (Callander et al., 2021; Cardenas-Marcelo et al., 2022; Delaney et al., [24, 30, 31]; Han [17, 26],. This work showed working conditions shifted across economic sectors but were felt hardest by the tertiary (service) sector, filled disproportionality by racialized women [17]. Much of this focused primarily on women’s increased domestic care responsibilities. Such increases and gendered division of care led in many cases to a reduction in women’s public working hours or heightened desires to leave the workforce during the pandemic [24, 26, 30, 31].

Highlighting the intersection of race and gender during the pandemic, a mixed method study conducted by Fan & Moen [30] in the United States revealed that Black women working remotely were more likely to experience a significant increase in work hours compared to white women and Black men [30]. Furthermore, when examining the intersection of gender and class, it was revealed that women without post-secondary degrees were more likely to experience decrease in work hours while highly educated women with advanced degrees or in managerial positions had a greater likelihood of experiencing a significant increase in work hours. This contrasts both less educated and more educated men or men in similar positions who were more likely to experience stable working hours during the pandemic [30].

Additionally, individuals historically marginalized including those working in precarious jobs such as sex work (Callander et al., 2021), and racialized women [24, 26] were found to have experienced disproportionately high financial losses during the pandemic. Interviews with twenty-one sex workers in the United States revealed such losses were partly due to lost working opportunities in efforts to reduce the spread of COVID-19 (Calendar et al., 2021). Sex workers at the intersections of race and gender are suggested to have experienced the most pronounced income losses (Callander et al., 2021). Additionally, findings reveal that disruptions to social connections had gendered and economic ramifications. Low income-women in Mexico were no longer able to access community support to meet financial and caregiving responsibilities [32].

The pandemic also appears to have amplified the safety risks associated with precarious work (Callander et al., 2021, Ndumbe-Eyoh et al., [26]. Thematic analysis of online conversations with practice, policy, research, and community leaders in Canada found that socioeconomic inequities among women and gender diverse individuals has contributed to the risk of contracting COVID-19 at work [26]. In addition, a safety risk for street based cis and transgender female sex workers facing financial insecurity during the pandemic reported in findings from Ndumbe-Eyoh et al. [26] during the pandemic, included being forced to meet clients in unfamiliar locations to replace income lost from closed venues.

#### Impacts of lockdown restrictions

A loss of community during the pandemic was a theme that ran across seven studies (Abreu 2021; Bibi [21, 28],; Calendar et al., 2022; Cardenas-Marcelo et al., 2022). One study found challenges related to loss of access to resources among lower income and racialized 2SLGBTQ + people [23]. Abreu et al. [23], identified the impact of the loss of support groups and community centers which served as places for affirmation and acceptance for many prior to the pandemic. This theme was also echoed by Calendar et al. (2022), that found a loss of community among sex workers compounded with a loss of clients and lack of protective policy left many in precarious economic positions and isolated. This study found that transgender, sexual minority, and low-income sex workers faced intersecting oppressions with barriers compounding on one another, shaping vulnerability to violence and marginalization (Calendar, 2022). Importantly, this study suggested the support of fellow sex workers in the sex work community to be crucial to sex worker wellbeing. Cardenas-Marcelo et al. (2022), also examined loss of community support, finding low-income women in Mexico faced heightened financial precarity during the pandemic as they were unable to rely on community supports available prior.

Additionally, Bettinger-Lopez et al. [28], considered the intersecting oppressions of gender and citizenship status faced by survivors of domestic violence in their Miami study utilizing focus groups and interviews. The authors found women without citizenship status were left especially vulnerable to domestic violence and abuse. Due to their precarious situation they were forced to make the difficult choice between living in an abusive situation, or leaving to seek aid, risking their health and well-being or deportation. In another study from the United States, Bibi [21] found that race, class, and gender intersect to shape experiences fleeing domestic violence.

#### Impacts on mental health

The impact of the pandemic on mental health was reported among four studies in this review [14, 15, 18, 27]. Low income, queer, nonwhite, and/or women, have faced increased rates of depression and anxiety [14, 15]. Additionally, two studies on Chinese Americans found that participants experienced increased rates of anxiety and depression during the pandemic [18, 27]. These studies came to contradictory conclusions as Litam & Oh [27] found middle aged Asian men faced the greatest rates of anti-Asian racism and thus greater rates of depression. Conversely, Oh & Min [27] found that Chinese American women faced the greatest amount of discrimination due to their intersecting identities.

## Discussion

The findings of this review demonstrate how intersectional theory and intersectional methods are informing pandemic-related research in North America. Intersectional studies and research that analyses multiple axes of difference show how pre-pandemic health care disparities have deepened throughout the pandemic. Much of this work focused on ways that the spread of dis-/mis-information (including the spread of conspiracy theories related to the virus) has hampered public health messaging. However, the impacts of these threats to public health have not been uniform. Data from the studies in this review show gender, younger age, and low levels of income and education leave people particularly susceptible to dis/mis-information Campos- [11]; Castillo & Laestadius, 2020; Fortune et al., [24].

This review found class as a significant factor among intersectional analysis of health care system disparities. Attention to the intersections of class shows that queer men [13] and women living in rural areas (Fortune et al., 2021) with low incomes were specifically shown to be less likely to access accurate information about the virus. While these studies also engaged with race, only results from Stockman et al. [20] show intersectional impacts of race and gender in their comparison of Black and white women’s knowledge of where to seek accurate information online. This study found that white women were more likely to be able to do so. A lack of focus on race is not reflected in broader, non-intersectional health research (see Ouyang et al., [6, 33, 34] for racial analysis without intersectional methods).

Stockman et al. [20] also conduct non-intersectional analysis of the pandemic. For example, analysis of race as an independent variable, detached from other social locations, found that those who had experienced racism were significantly more likely to be vaccine hesitant [35]. However, intersectional work does not simply add further data. Instead, intersectional analysis offers a lens through which to understand how health disparities shifted and deepened in unique ways. Barriers to good health were never uniform and pandemic impacts have deepened pre-existing inequities. Intersectional methods, like centralizing oppression in analysis, also turn attention to structure. This enables researchers to develop solutions that extend beyond individuals and engage at the systemic level.

Similar to health care, disparities in social service access existed prior to the pandemic. In this review, both themes were separated as included studies addressed specific or targeted social programs, whereas those concerned with health care were organized more by topic (for example access to health information and vaccine hesitancy). Class was also central through the studies with attention to social service barriers [14, 24]. Bettinger-Lopez & Montes (2022) assert that rates of GBV increased during the pandemic. This claim is supported by evidence that measurements of intimate partner violence (IPV) (including calls to support lines and police reports) increased in China, Spain, Italy, the United Kingdom, France, and Canada [36]. What Bettinger-Lopez et al. [28] add to this discussion, is a fuller understanding of those impacted by IPV. For example, their attention to oppression and comparison highlights ways that dependency often makes people vulnerable to GBV, including IPV. The authors work in Florida shows how economic dependency and legal precarity shape not only vulnerability to and experiences with GBV, but also access to services and recourse following harm.

Pandemic lockdown restrictions limited access to services in ways that may appear equal. A common phrase throughout the first two years of the pandemic, “We’re all in the same boat” signaled this understanding of service loss. However, intersectional analysis of service access, (which as we report has focused on GBV, worker safety, education, and youth programming), offers a different view. Instead of all being in the same boat, some commentators explain we are all in the same storm but in very different boats ranging from luxury yachts with massive crews, to exposed and isolated sailboats [37].

This review also found that intersectional analysis of changes to work and working conditions has considered these changes across three fronts: increased gendered domestic care responsibilities, financial losses, and exacerbated job safety risks. None of these working conditions are true changes or departures from pre-pandemic labour organization. Rather, they are all connected to prior systems of gendered and racialized labour exploitation (Callander et al., 2021; Cardenas-Marcelo et al., 2022; Fortune et al., [24, 26]. Here we see convergence between current intersectional research and Marxist feminist accounts of labour relations in North America. This may be significant as recent critiques of intersectionality charge the theory with losing its critical potential [38, 39]. Such critique has called for the explicit critical grounding of intersectionality theory through engagement with Marxist feminism [39, 40]. Others have approached such a convergence in reverse, asserting it is imperative we adopt an intersectional understanding of Marxism to extend analysis of economic relationships within specific economic conditions [41, 42].

Building on the findings of this review, the compounded vulnerabilities experienced by marginalised workers during the pandemic, particularly racialized women in precarious sectors, illustrate the intersectional nature of labour exploitation. Intersectionality theory highlights how overlapping identities such as race, gender, and socioeconomic status shape experiences of inequity [1, 2]. Marxist feminist critiques further contextualise these inequities within capitalist labour systems that systematically undervalue racialized and feminised labour [38, 40].

For example, women of colour working in care and service sectors faced heightened risks of job insecurity, unsafe working conditions, and increased caregiving responsibilities at home (Callander et al., 2021; Fortune et al., [24]. These findings align with Marxist feminist critiques of how capitalist structures rely on and exploit the undervalued and unpaid labour of marginalised groups, exacerbated under crises like the pandemic. Future integration of intersectionality and Marxist feminism could provide a robust framework for addressing these systemic issues through policy and practice.

This review does not add to these theoretical debates. We cannot offer support of approach in either direction. Marxism may be a tool through which to re-anchor intersectionality to critique. On the other hand, it may be more appropriate to see intersectionality as a means through which to refine Marxist analysis. Either way, our review found evidence that these theoretical lenses are being adopted alongside one another, even if they are being done without explicit use of Marxist thought. Future work which can bring these theories together in more explicit ways may yield both greater insight into the pandemic’s impacts, as well as a response to critiques of both theories. Our findings do suggest that McCall’s [43] claim that intersectional methodological approaches had yet to deal with the issue of complexity remain unresolved nearly two decades later.

In their analysis of employment conditions during the pandemic Andrea et al. [44] investigate the intersections of race, gender, education, and age on worker wellbeing in the United States. They found that the pandemic exacerbated previous economic and social inequities and proposed that improvements to long term care must address devalued racialized and gendered labour.

Evidence from Andrea et al. [44] and the studies included in our review (Callander et al., 2021; Delaney et al., [24, 26, 30, 31], support a need for a more intersectional policy response to pandemic effects. This is significant as other work has critiqued Canada for failing to commit to gender-based analysis in research and policy [4], and for lacking adequate race-based health data [5]. Recently, the federal government has committed to gender-based analysis in policy, gender training for civil servants, and the collection of race aggregated data moving forward.

Literature included in this review shows how lockdowns interrupted community connections. Implications of loss of community were felt inequitably. For some, particularly those who are marginalized at the intersections of gender, sexuality, and citizenship and/or who work as sex workers, loss of community came with crippling loss of emotional and financial support as well as access to other resources. This left some, like those with precarious status and those in the sex work industry, uniquely vulnerable to gender-based violence [28]; Calendar et al., 2022).

Our findings here add to non-intersectional research about community ties during pandemic restrictions by foregrounding the ways that interruptions to community connections shaped access to resources which for some, shaped their life chances. Other work has focused more on the impacts of community loss and lockdown isolation on mental health, without considering resource implications [45, 46]. This is not to say attention to interactions between community ties and mental health is not important. Rather, pandemic impacts to mental health also featured prominently within this literature. Links within this review are reflected in broader research as community belonging was found to be a strong predictor of mental wellness among Indigenous people [47].

A common line across themes was that the pandemic deepened already existing social and economic inequities. The findings of this review demonstrate that the COVID-19 pandemic exacerbated pre-existing social and economic inequities, particularly for marginalised groups situated at the intersections of race, gender, class, and other social identities. For example, racialized women in precarious employment and low-income mothers experienced heightened financial insecurity, restricted access to social services, and increased caregiving responsibilities, which deepened their vulnerabilities during the pandemic (Callander et al., 2021; Fortune et al., [24, 26]. Moving forward, it is essential for researchers and policymakers to adopt intersectional approaches that account for the complex realities faced by marginalised populations. Future studies should prioritise the collection and analysis of disaggregated data to better identify and address compounded inequities. Pandemic recovery efforts must include targeted interventions that prioritise groups disproportionately affected by the crisis, such as racialized women in the care and service industries. Moreover, strengthening community-based resources and fostering a sense of belonging will be critical in building resilience and mitigating the long-term effects of the pandemic on vulnerable populations. These steps are necessary to address the structural inequities that shaped pandemic experiences and to ensure more equitable outcomes in future public health crises.

Similarly, this was also true of impacts to mental health. Those who were already marginalized along multiple axes were found to have faced increased rates of depression and anxiety [14, 15]. Significantly, we found that work centralizing intersections of race was limited to Chinese Americans [18, 27]. There is a lack of attention to other racialized groups and to other forms of racism within the literature. This is not reflected in broader, non-intersectional research. For example, Chae et al. [48], found racism in the United States connected to mental health challenges among Asian and Black Americans. Liu and Modir [7], found similar evidence among Asian, Black, and Latinx communities.

The two studies about the intersections of race in relationship to mental health offered contradicting claims that either men [18], or women [27], were more vulnerable to poor mental health stemming from anti-Asian racism. Seens et al. [49] argue that women and gender-diverse people, especially those with children, suffered greater mental health burdens during the pandemic than others. Other, non-intersectional work has also asserted women and gender diverse people are more at risk of poor mental health stemming from pandemic impacts [50]. Of additional note, included literature shone attention on university students’ mental health [14, 15], but attention to primary and secondary students/children is lacking. This may be an interesting direction for further research.

## Limitations

This scoping review is limited to research conducted in North America, which may restrict generalisability to other contexts. Relevant studies might have been missed due to publication bias or the use of English-only sources. Future research should expand geographic scope and include non-English studies.

## Conclusion

This scoping review found that intersectional research methods are increasingly utilised in COVID-19 pandemic research in North America. Our findings highlight how the pandemic deepened existing inequities, particularly for marginalised populations at the intersections of race, gender, and class. These inequities underscore the need for public health policies informed by intersectional frameworks. Disaggregated data collection should become standard practice to identify inequities, and targeted interventions—such as workplace protections for racialized women in precarious jobs are critical to addressing compounded vulnerabilities.

These findings can be considered together with our analysis of intersectional research methods, which was also conducted using this data set but has been developed as an independent paper. Furthermore, these findings suggest the value of a more explicit convergence between intersectionality and Marxist feminist accounts of labour relations in North America. For example, public health systems can operationalise these frameworks by investing in equitable labour protections and embedding community-based participatory approaches into policy development. These steps can address structural inequities while fostering resilience and inclusivity in public health systems.

Future research should explore how these theoretical frameworks can be systematically embedded into public health policies and interventions. Such efforts will ensure that the inequities exposed by the pandemic are not repeated in future crises.

## Data Availability

The data that support the findings of this study are available from the corresponding author, [B.S.], upon reasonable request.

## Footnotes

1 Reason for Exclusion: Duplicate. Does not meet the inclusion criteria (use of intersectionality theory or intersectional analysis of two or more axes of identity; focus on COVID-19; in North America). Adapted from: Page et al. [51]. The PRISMA 2020 statement
